# An *in silico* osmotic pressure approach allows characterization of pressure–area isotherms of lipid monolayers at low molecular areas†

**DOI:** 10.1039/d2sm01419j

**Published:** 2023-04-10

**Authors:** Janak Prabhu, Akhil Pratap Singh, Stefano Vanni

**Affiliations:** a Department of Biology, University of Fribourg, Chemin du Musée 10 1700 Fribourg Switzerland stefano.vanni@unifr.ch

## Abstract

Surface pressure–area isotherms of lipid monolayers at the air–water interface provide essential information about the structure and mechanical behaviour of lipid membranes. These curves can be readily obtained through Langmuir trough measurements and, as such, have been collected for decades in the field of membrane biochemistry. However, it is still challenging to directly observe and understand nanoscopic features of monolayers through such experiments, and molecular dynamics (MD) simulations are generally used to provide a molecular view of such interfaces. In MD simulations, the surface pressure–area (*Π*–*A*) isotherms are generally computed using the Kirkwood–Irving formula, that relies on the evaluation of the pressure tensor. This approach, however, has intrinsic limitations when the molecular area in the monolayer is low (typically < 60 Å^2^ per lipid). Recently, an alternative method to compute *Π*–*A* isotherms of surfactants, based on the calculation of the three-dimensional osmotic pressure *via* the implementation of semipermeable barriers was proposed. In this work, we investigate the feasibility of this approach for long-chain surfactants such as phospholipids. We identify some discrepancies between the computed values and experimental results, and we propose a semi-empirical correction based on the molecular structure of the surfactants at the monolayer interface. To validate the potential of this new approach, we simulate several phosphatidylcholine and phosphatidylethanolamine lipids at various temperatures using all-atom and coarse-grained force fields, and we compute the corresponding *Π*–*A* isotherms. Our results show that the *Π*–*A* isotherms obtained using the new method are in very good agreement with experiments and far superior to the canonical pressure tensor-based method at low molecular areas. This corrected osmotic pressure method allows for accurate characterization of the molecular packing in monolayers in various physical phases.

## Abstract 2

We present an improved computational methodology to characterise surface pressure–area (*Π*–*A*) isotherms for Langmuir monolayers. Our approach is particularly suited to the study of *Π*–*A* isotherms at low molecular areas.

## Graphical abstract

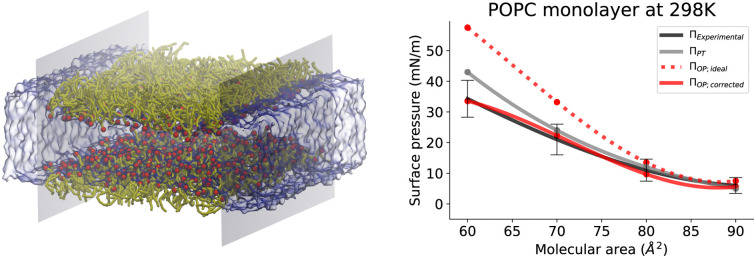

## 1 Introduction

Lipid monolayers are vital structures that play an essential role in a plethora of biological functions.^1–5^ These include the monolayer of a pulmonary surfactant, which spreads as a monomolecular layer on the alveolar liquid to forbid the collapse of the alveoli,^1,2^ and that of the tear film lipid layer in the eyes, which allows the non-polar fluid to spread quickly between the eyes while blinking.^3^ Moreover, lipid monolayers are also used as model membrane systems to study the membrane structure and protein–membrane interactions as they are relatively easy to analyze compared with lipid bilayers.^6,7^ As a consequence, the investigation of lipid monolayers, including the process of their spreading and formation at the air–water interface, has received significant interest over the last few decades.^8–12^

A key thermodynamic parameter that recapitulates the interfacial interactions in lipid monolayers at the air–water interface is the surface pressure. Additionally, the surface pressure–area (*Π*–*A*) isotherm of lipid monolayers also provides information on their phase behaviour,^11,13^ including liquid condensed (LC) phase, liquid expanded (LE) phase, phase coexistence (LC–LE), and monolayer collapse. However, such experimental studies have limitations in providing direct information related to the dynamics of lipids and their nanostructures. To overcome this limitation, molecular dynamics (MD) simulations have demonstrated great promise, providing atomic-level descriptions of complex systems and explaining their nanoscopic features beyond the limitations of existing experimental methods.^14–17^

Many all-atom (AA) and coarse-grained (CG) MD simulation studies have investigated lipid monolayers by obtaining their *Π*–*A* isotherms and other structural properties.^12,18–21^ Classically, to compute the surface pressure using MD simulations, two sets of simulations are required. First, one needs to determine the surface tension of the air–water interface (*γ*_w_) at the specified temperature. Next, one needs to obtain the surface tension of the air–monolayer–water interface (*γ*_m_). The monolayer surface pressure is subsequently obtained as the difference between these two surface tension values, *γ*_w_ and *γ*_m_. This process is repeated at different lipid—surface coverages (*i.e.*, at different values of molecular areas at the air–water interface) to obtain a complete *Π*–*A* isotherm. To determine both *γ*_w_ and *γ*_m_, the diagonal values of the pressure tensor (*P*_*xx*_, *P*_*yy*_ and *P*_*zz*_) are required, and the surface tension is estimated using the Kirkwood–Irving formula.^22^ With this method, at large molecular areas MD simulations provide satisfactory explanations for physicochemical processes of lipid monolayers by reproducing the phase behaviour of monolayers.^16,19,23^ However, at low molecular areas, typically below 60 Å^2^, inadequate reproducibility of thermodynamic properties,^19–21,24^ including that of surface pressure–area isotherms, has been observed, even when MD simulations are carried out with accurate all-atom (AA) models. For example, Javanainen and co-workers^24^ employed a four-point OPC4 water model^25^ to reproduce experimental surface pressure−area isotherms for DPPC and POPC monolayers with quantitative accuracy. However, they also found discrepancies when the lipid area was less than 60 Å^2^. Furthermore, Zhu *et al.*^26^ also calculated the *Π*–*A* isotherms for the same monolayers by performing CG-MD simulations and they were unable to correctly capture the isotherms at a lower molecular area.

Recently, De Souza *et al.*^27^ proposed a novel method for calculating the surface pressure of surfactants. The authors demonstrated that the 3D osmotic pressure method presented by Luo and Roux^28^ could be used in combination with MD simulations to calculate the surface pressure of zwitterionic and ionic surfactant monolayers. This is achieved by setting virtual walls to confine the monolayers to a particular region. The virtual walls behave as an osmotic membrane, where the water molecules are allowed to move freely, but the surfactant molecules are pulled back by the force exerted by the wall. In this work, we extend this approach to long-chained surfactants such as phospholipids, to obtain complete *Π*–*A* isotherms for lipid monolayers at the air–water interface. We found that this method, albeit superior to the classical pressure-method, remains unable to accurately describe the behaviour of monolayers at low molecular areas. To address this issue, we propose a semi-empirical correction based on the nanoscopic structure of the water–monolayer–air interface. By implementing this approach, we reconstruct the *Π*–*A* isotherms and we find excellent agreement with experimental observations for lipid monolayers, including at low molecular areas.

## 2 Methods

### 2.1 Molecular dynamics simulations

#### 2.1.A All-atom simulations

The AA simulations in the current work were carried out with the Nanoscale Molecular Dynamics (NAMD) software.^29^ The CHARMM36 lipid FF^30^ optimized for the OPC4 water model as implemented by Javanainen *et al.* was utilized to run AA-MD simulations. The lipid monolayers (POPC) without water were first generated with the CHARMM-GUI package.^31,32^ The OPC4 water box was created using PACKMOL.^33^ Finally, virtual molecular dynamics (VMD) was used to solvate the monolayers.^34^ The AA-MD simulations were performed in the NVT ensemble, and the Langevin thermostat was used to control the temperature. The integration time step size was set to 2 femtoseconds. All non-bonded interactions were cut-off at 12 Å. The Particle Mesh Ewald (PME) algorithm^35^ was implemented for long range electrostatic interactions.

#### 2.1.B Coarse grained simulations

**Fig. 1:**
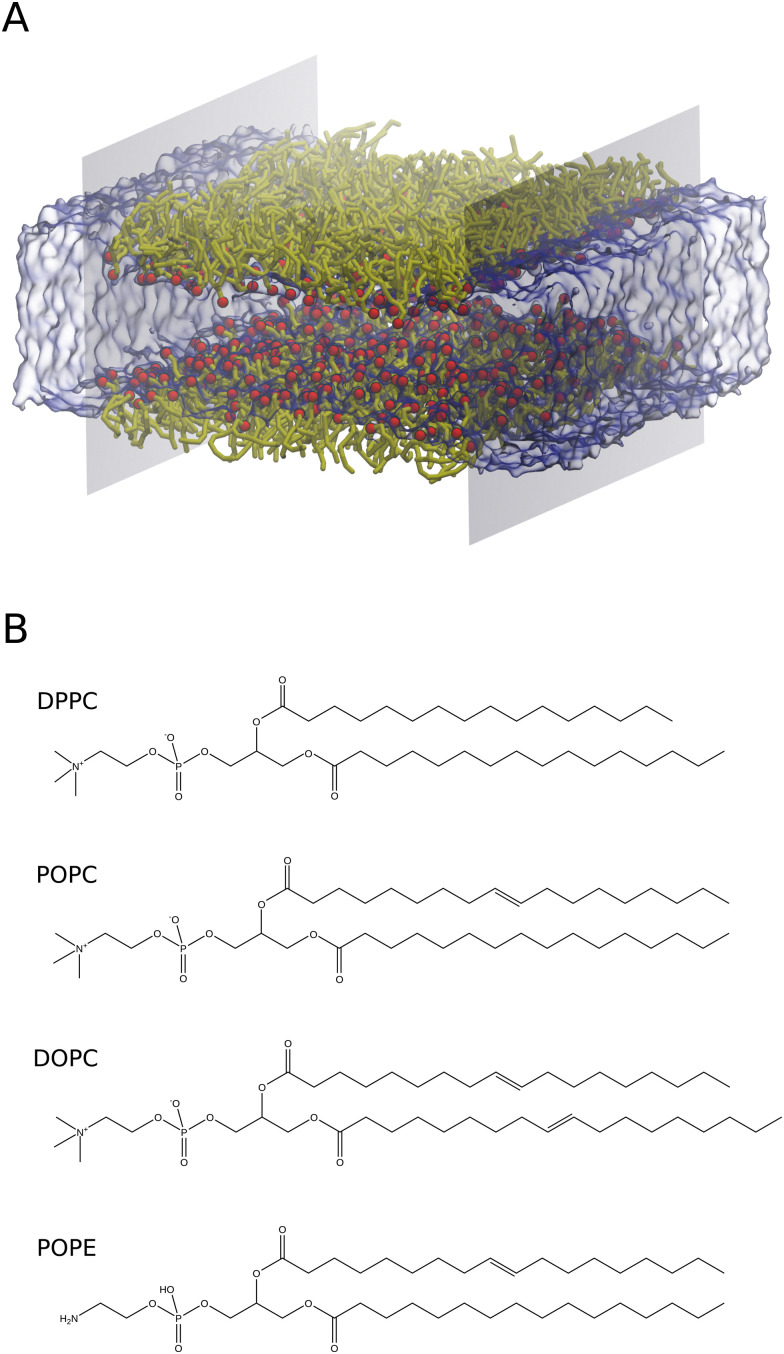
(A) Spatial arrangements of the lipid monolayer with virtual walls implemented to obtain the surface pressure by an osmotic pressure method. Colour scheme: red for lipid head groups, yellow for lipid tails, ice blue for water and grey for virtual walls. (B) Molecular structures of DPPC, POPC, DOPC and POPE lipids.

All CG-MD simulations were carried out with Large-scale Atomic/Molecular Massively Parallel Simulator (LAMMPS) simulation software.^36^ To create CG monolayers, different AA lipid structures were obtained from the CHARMM-GUI package (Fig. 1a). PC and PE lipids with different acyl chains (DOPC, POPC, DPPC, and POPE) were used in this study (Fig. 1b). These systems were then transformed from AA to CG representations, compatible with the surface property fitting coarse-grained force field (SPICA FF),^38,39^ using the CG-it tool [https://github.com/CG-it/CG-it]. Finally, the CG-lipid monolayer systems were built with PACKMOL. All simulations were carried out in the NVT-MD ensemble unless specified otherwise, and the Nosé–Hoover thermostat^37^ was implemented to control the temperature for the simulations. The integration time step size was 10 femtoseconds. All non-bonded interactions were truncated at a cut-off distance of 15 Å. The PME algorithm implemented for SPICA FF in LAMMPS was used for long-range electrostatic interactions with a real space cut-off of 15 Å. Periodic boundary conditions (PBC) were applied in all directions for all the simulations. All snapshots were rendered using VMD.

#### 2.1.1 Coarse grained model

The SPICA coarse-grained molecular mechanics (MM) potential energy function^38,39^ of the following form was adopted to delineate the various interaction points in the lipid monolayer systems.
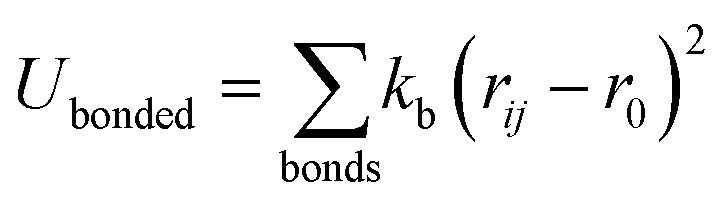

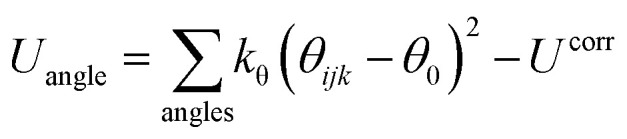

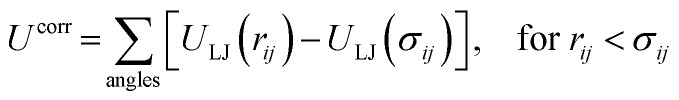

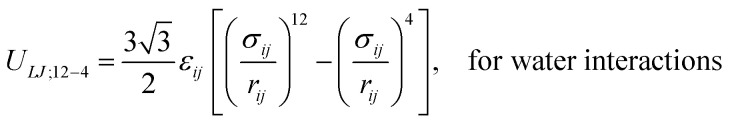

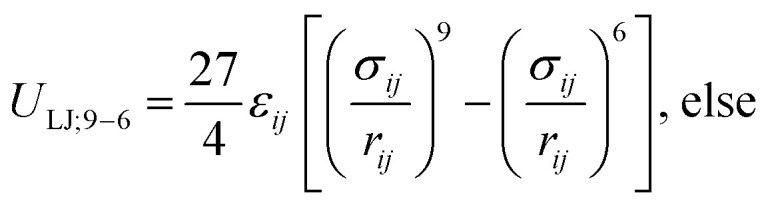
1
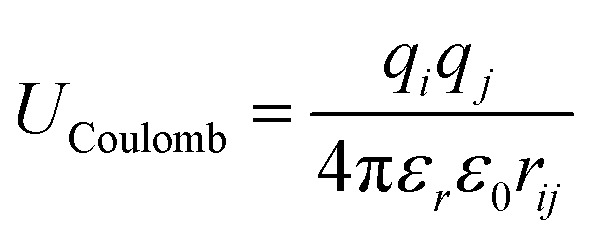


#### 2.1.2 Pressure tensor systems

##### 2.1.2.A All-atom systems

AA systems were generated with 128 lipids (64 lipids per interface) in the simulation box. The polar head groups of lipids are oriented towards the water phase as shown in Fig. 1A and Fig. S1 (ESI†). The water slab was covered with lipids from both sides in the *z*-direction. The ratio of lipids to water molecules was 1 : 30. This creates a water slab of around 40 Å in the *Z* direction, to avoid lipid headgroup–headgroup interactions across the water region. A vacuum region of 30 Å in length along the *z*-direction was created to prevent lipid tail–tail interactions due to PBC conditions. Notably, these systems were built explicitly for each molecular area to calculate *Π*–*A* isotherms. The air–monolayer–water interface simulations were carried out until the surface tension values converged. Furthermore, we also performed CG simulations to calculate the air–water surface tension using a water slab with 4000 water molecules. To do so, we used the same simulation conditions as described in Section 2.1.A.

##### 2.1.2.B Coarse grained systems

All monolayer systems with different lipids were created by placing 256 lipids (128 lipids in each interface) in the simulation box. The systems were then set up following the same process as explained above for AA-simulations. The conditions to carry out all CG-MD simulations are described in Section 2.1.B. Table S1 (ESI†) lists the information regarding each system.

#### 2.1.3 Osmotic pressure systems

The initial configurations of the monolayers have been prepared in the same manner as described in a previous section (Section 2.1.2). Each leaflet of lipids was spread over a variable area in the *xy*-plane as we implemented the movable virtual walls in the *x*-direction. Varying the distance between walls is necessary to reach different packing conditions (molecular area), since the number of lipids was kept constant. Notably, the virtual walls along the *x*-direction were imposed by implementing a Tcl script^27^ and the LAMMPS fix indent procedure, for the AA and CG simulations, respectively. AA and CG simulations were carried out for 50 and 100 ns, respectively, at a given molecular area conformation, where the virtual walls were held static. Details regarding system setup and position of the virtual wall for each lipid monolayer are provided in Table S2 (ESI†).

### 2.2 Surface pressure calculations

#### 2.2.1 The classical pressure tensor approach

Using the classical pressure tensor approach, known as the pressure tensor-based method, we computed the surface tension by using the diagonal values of the pressure tensor (*P*_*xx*_, *P*_*yy*_ and *P*_*zz*_) implementing the Kirkwood–Irving method,^22^ as follows:2
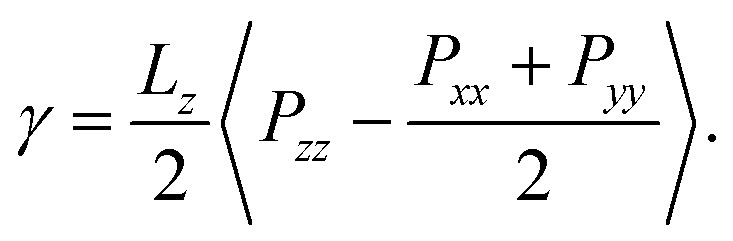
where, *L*_*z*_ is the length of the simulation box in the *z*-direction, and 〈…〉 is the ensemble average. Furthermore, the surface pressure (*Π*) of the monolayers was calculated using the following equation:

3 *Π* = *γ*_w_ − *γ*_m_

where the air–water interface surface tension is given as *γ*_w_ and the air–monolayer–water interface surface tension is given as *γ*_m_.

#### 2.2.2 The osmotic pressure approach

We computed the surface pressure by implementing the osmotic pressure-based methodology as follows: two virtual walls were introduced in the *x*-direction as semipermeable membranes (Fig. 1A). Notably, these walls work like mobile barriers of a Langmuir trough. The virtual walls allow the flow of water freely across them and restrict the lipids to the region between the walls, imitating a membrane. This procedure is carried out by allowing the walls to apply flat-bottom planar restraints with a force constant *k* to the lipids. This allows the holding of the lipid molecules in the specified region. The force exerted by these walls during the simulations corresponds to the osmotic force 〈*F*_wall_〉. The osmotic force was calculated using the following relationship, proposed by Luo *et al.*^28^4
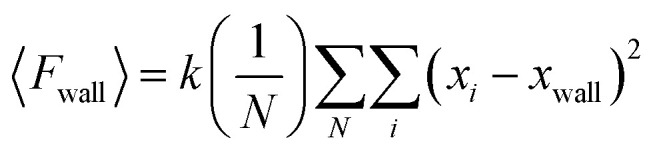
where, |*x*_*i*_| > |*x*_wall_|, *N* is the number of saved frames of the trajectory data and i is the index of the molecular bead. In our simulations, we have implemented *k* = 20 kcal mol^−1^ Å^−3^, which is neither too restrictive nor too weak. It is worth mentioning that changes in force constant *k* did not change the outcome, consistent with previous studies.^27^ Notably, the *x*-coordinate trajectories of the lipid beads were saved at each picosecond to calculate the osmotic force.

Subsequently, 〈*F*_wall_〉 was implemented to calculate the 2D surface pressure, *Π*_*ideal*_, as5
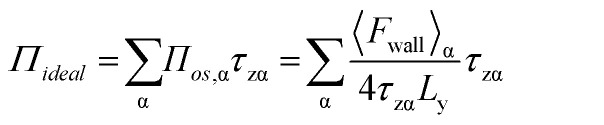
where, *Π*_*os*,α_ is the species' contribution to the 3D osmotic pressure. *τ*_zα_ is the depth towards the sub-phase (*z*-direction) for the α species and *L*_y_ is the length of the box in the *y*-direction. The expression is divided by a factor of 4 as two virtual walls have intersections with the air–monolayer–water interface.

If the depth of the surface region *τ* is considered to be one molecule thick, eqn (5) can be rewritten as follows, without the sum over species6
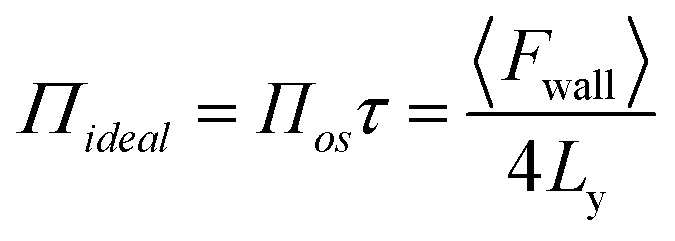
where, *Π*_*os*_ is the total 3D osmotic pressure.

The derivation by De Souza *et al*. follows eqn (6) from Adamson.^40^ There, surface pressure *Π*_*ideal*_ is the osmotic pressure *Π*_*os*_ times the depth of the surface region *τ* where the force is exerted. It is worth mentioning that they used an ideal approach to treat the surface region as a kind of solution in which the depth of the surface region (interfacial thickness) is the same as the surfactant molecular length. However, previous studies have demonstrated that monolayers at the liquid–vapor interface are complex systems and have an inhomogeneous transition surface zone that consists of the water molecules, head groups, and chain segments of molecules.^41,42^ More importantly, this inhomogeneous transition zone fluctuates for the monolayer gas-to-liquid transition as molecule penetration depth varies for various molecule concentrations at the surface. Therefore, using an ideal definition of the surface zone, such as considered by De Souza *et al*., for a monolayer of long-chain molecules supported on water would be ambiguous.

**Fig. 2:**
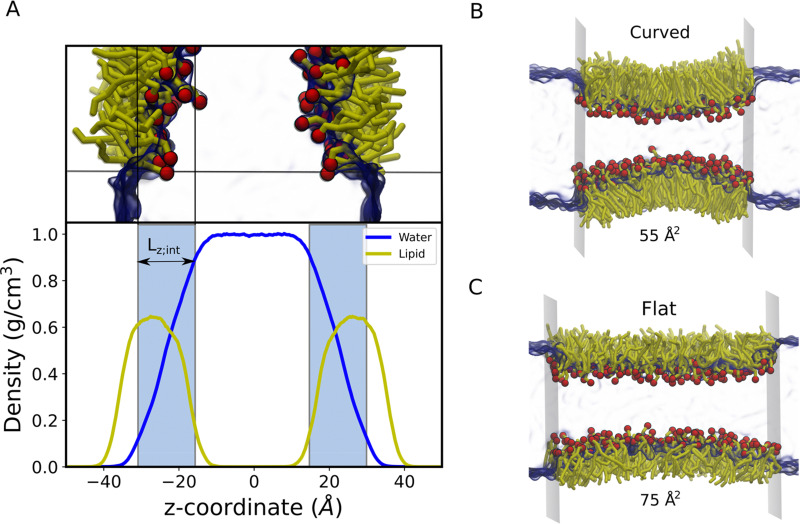
(A) Partial density profiles for water (blue solid line) and lipids (yellow solid line) along the monolayer normal to obtain the interfacial thickness (*L*_z;int_) to calculate in 3D the osmotic pressure. The regions corresponding to *L*_z;int_ are represented by ice blue. Notably, *L*_z;int_ represents the region where the water density drops from 90% to 10% of the maximum density. The inserted MD snapshot of the lipid monolayer illustrates the corresponding region of *L*_z;int_ more directly. The arrangement of lipid monolayers at (B) the low molecular area (55 Å^2^) and standard molecular area (75 Å^2^). Colour scheme: red, lipid head groups; yellow; lipid tails; blue, interfacial water. For clarity, water is given in translucent blue.

Popielawski and Rice^42^ have proposed a generalized regular solution model of a liquid-supported monolayer of long-chain molecules. They determined the dependence of surface pressure on the effective chain length (*L*) in the surface layer. Furthermore, they also demonstrated that the relationship between *L* and surface coverage is such that *L* is greatest at infinite dilution and gradually falls as the surface coverage increases. By using the approximation of their model and other available knowledge,^41–46^ we now introduce an approximate expression to obtain the surface pressure of the monolayer from gas to liquid transition. Thus, a monolayer may be considered to exert surface pressure (*Π*_*real*_) more significantly using the following expression,

7 *Π*_*real*_ = *Π*_*ideal*_*ξ*

where, *ξ* is a semiempirical coefficient that is treated as an adjustable parameter. Notably, *ξ* is greatest (close to 1) when the surfactants are widely spread out (essentially flat; see Fig. 2C) and becomes lower than 1 when the surfactants tend to pack and cover more interfacial area (Fig. 2B). It is noteworthy that the definition of *ξ* is in accordance with Popielawski and Rice's monolayer model. Here, we opted to define *ξ* as the inverse ratio between the interfacial depth of the inhomogeneous surface zone (*L*_z;int_, Fig. 2A) at a given molecular area, and the interfacial depth at very low surfactant surface coverage (asymptote of *L*_z;int_ for infinite dilution). The details for the calculation of *ξ* are provided in the ESI.† In detail, *L*_z;int_ is defined as the region where the density of water along the direction normal to the interface drops from 90% to 10% of the maximum density,^18,47,48^ as shown in Fig. 2A. The value of *L*_z;int_ varies with the compression or expansion of monolayers as it depends on the concentration of molecules on the surface. Therefore, we calculated the value of *L*_z;int_ at each molecular area configuration by using the density profile of the sub-phase and monolayers in the *z*-direction. In all the simulations, density profiles were calculated using trajectory files with the implementation of the Tcl script^49^ in VMD. Notably, to allow the water to flow across the virtual walls freely, we consider an extra region of water molecules without lipids (clean interface) beyond the virtual walls equal to 18 Å in both direction along the *x*-axis for each lipid monolayer system, like in the previous report.^27^

## 3 Results & discussion

**Fig. 3:**
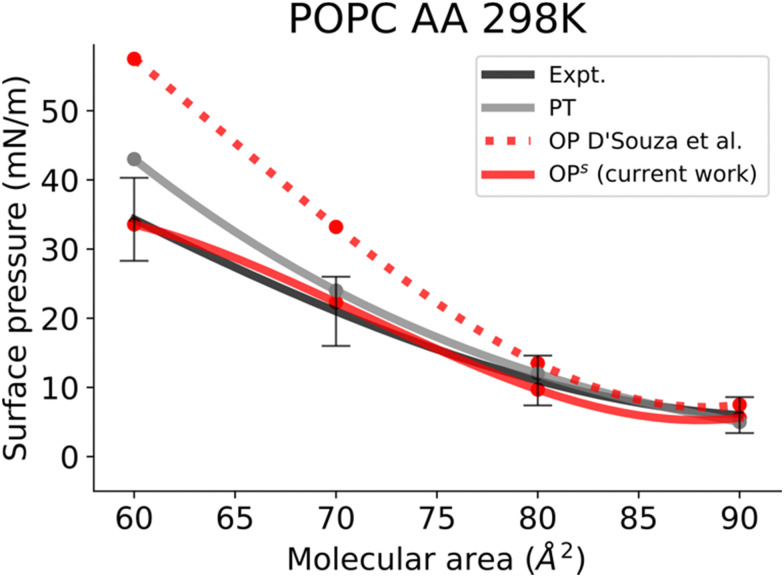
Surface pressure–molecular area isotherms of AA POPC lipid monolayers at an aqueous sub-phase at 298 K. Label scheme: Expt. for the experimental *Π*–*A* isotherm, PT for the *Π*–*A* isotherm obtained using the pressure tensor method and OP for the *Π*–*A* isotherm obtained by the osmotic pressure method by D’Souza *et al.* OP^s^ for the *Π*–*A* isotherm obtained by the osmotic pressure method with semi-empirical correction (current work). Experimental error bars are included.

In this work, we simulated several phosphatidylcholine (PC) and phosphatidylethanolamine (PE) lipids (Fig. 1B) at various temperatures and obtained their *Π*–*A* isotherms to validate the osmotic pressure approach. To evaluate our approach to obtain the surface pressure isotherm, firstly we performed AA-MD simulations on the POPC monolayer, with the OPC4 water model of CHARMM36 FF. Our choice was based on the observation that the OPC4 water model has shown excellent reproduction of the water–air surface tension of water, and that this parameter significantly impacts the reproduction of the surface–pressure area isotherm utilising the pressure-tensor approach of calculating the isotherm.^24,50^ The comparison of *Π*–*A* isotherms obtained by osmotic pressure and pressure tensor methods with OP4 water model, at 298 K, with experimental measured^12,51–53^ are shown in Fig. 3. For experimental *Π*–*A* isotherm data for POPC, we plotted the average value resulting from various reports,^12,51,52^ with the confidence interval given as the standard deviation as shown in Fig. 3.

It is evident from this figure that while both the pressure tensor method (PT) and the osmotic pressure methodology (OP) can reproduce the experimental data at large molecular areas, they both struggle to do so at low molecular areas (area per lipid < 65 Å). We reasoned that, for the osmotic pressure methodology, the origin of this discrepancy could originate from the original assumption made by De Souza *et al*. to assume that the depth of the surface region (interfacial thickness) is identical to the surfactant molecular length (see the Methods section for a detailed description). To alleviate this problem, we propose an empirical correction term such that it is close to 1 when the surfactants are very spread out (essentially flat), while becoming lower than 1 when the surfactants tend to pack and cover more interfacial area. Based on empirical and theoretical considerations (see the Methods section), we define this coefficient as the inverse ratio between the interfacial depth of an inhomogeneous surface zone (water density values between 10 and 90%) at a given molecular area, and the same value at very low surfactant surface coverage (asymptote for infinite dilution) (Fig. 2A). By applying this correction, the modified osmotic pressure methodology (OP^s^) can correctly reproduce the experimental isotherm for POPC using AA simulations (Fig. 3).^38^

To further check the validity of our approach, we next opted to compute *Π*–*A* isotherms for various phospholipids using a CG Force field. Our choice was motivated by the fact that CG-MD simulations enable us to compute this property while requiring fewer computational resources and costs. For CG-MD simulations, we have employed the SPICA-FF as it shows an excellent reproduction of the air–water interface over a wide range of temperatures relevant to physiological processes.^38,54^ To ensure robustness of our approach further, PC and PE lipid monolayers were simulated over a range of molecular areas to obtain the *Π*–*A* isotherms. Furthermore, we compare these *Π*–*A* isotherms with those calculated through the pressure tensor-method and measured experimentally.

### 3.1 Surface pressure–area isotherms of POPC and DOPC

**Fig. 4:**
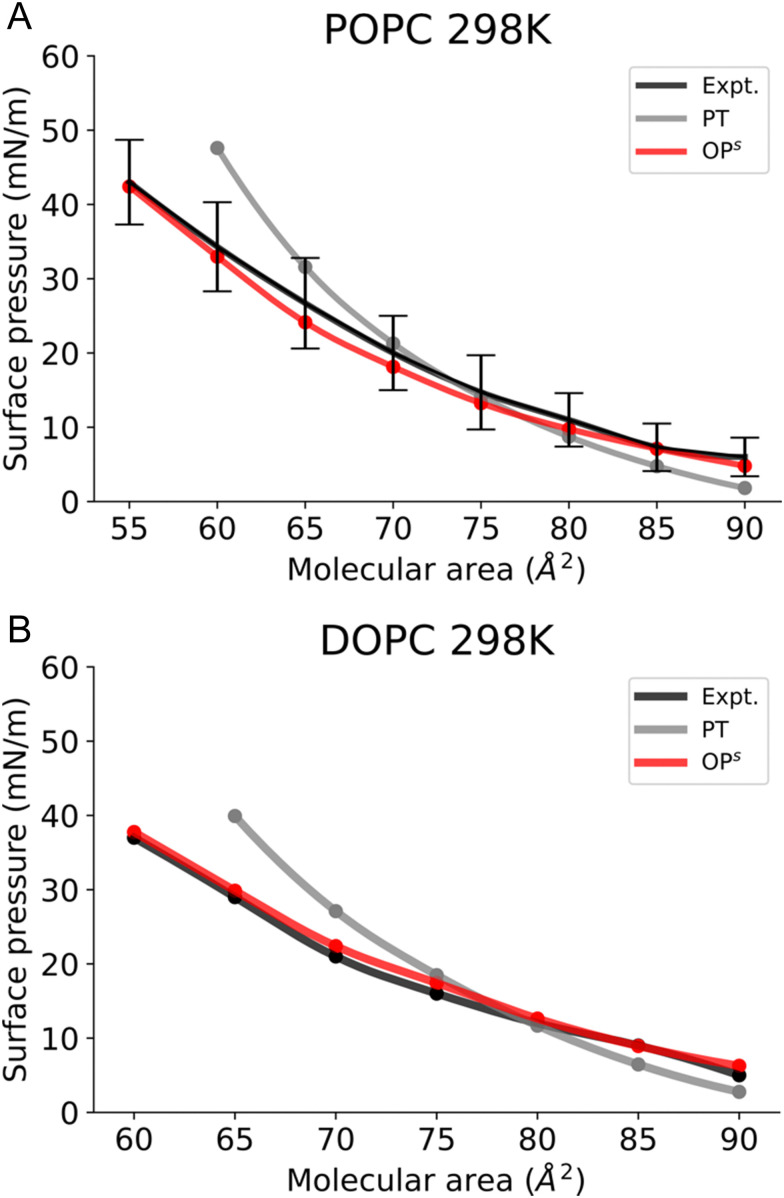
Surface pressure–molecular area isotherms of (A) POPC, (B) DOPC lipid monolayers at the aqueous sub-phase at 298 K. *Label scheme*: Expt. for the experimental *Π*–*A* isotherm, PT for the *Π*–*A* isotherm obtained using the pressure tensor method and OP^s^ for the *Π*–*A* isotherm obtained using the current osmotic pressure method. Error bars are included.

To validate our new method, we first simulated monolayers consisting of lipids with a melting point (*T*_m_) below the room temperature, POPC and DOPC (Fig. 4). We first tested whether using the osmotic pressure method to obtain the *Π*–*A* isotherms, *i.e. via* compression (blue) or expansion (red) affects the final *Π*–*A* isotherms. Notably this is not the case, as both curves are identical (Fig. S2, ESI†).

Next, we compared our *Π*–*A* isotherms with the available experimental data. The experimentally accessible and reliable data for POPC is provided at molecular areas ≥ 55 Å2 and for DOPC at molecular areas ≥ 60 Å^2^ (Fig. 4). The choices for the molecular area are slightly higher than the monolayer equilibrium collapse area of these lipids (51 Å^2^*vs* 54 Å^2^, respectively).^55^ Our simulation results show that POPC and DOPC monolayers reside in the LE phase at 298 K (Fig. S3, ESI†) and remain stable above the equilibrium collapse pressure (∼40–45 mN m^−1^), consistent with experimental reports. Fig. 4A shows that, in the case of POPC, the osmotic pressure-based methodology accurately reproduces the experimental *Π*–*A* isotherm. In contrast, the pressure tensor method cannot reproduce the isotherm below the molecular area of 65 Å^2^.

A similar observation is made in the case of DOPC, where the osmotic pressure-based method reproduces well the experimental curve; however, the pressure tensor-based technique does not reproduce the isotherm below 70 Å^2^, as shown in Fig. 4B. It is also worth mentioning here that we could not determine the surface pressure at small molecular areas (<60 Å^2^) with the pressure tensor method as the system is unstable (Fig. S4, ESI†). Thus, calculation of the *Π*–*A* isotherms using osmotic pressure allows us to accurately reproduce experimental curves for fluid bilayers, such as POPC and DOPC, up to low molecular areas.

### 3.2 Surface pressure–area isotherms of DPPC and POPE

Subsequently, we investigated the use of this method for more complex monolayers, with coexisting LE and LC phases. To do so, we focused on DPPC monolayers at different temperatures.

**Fig. 5:**
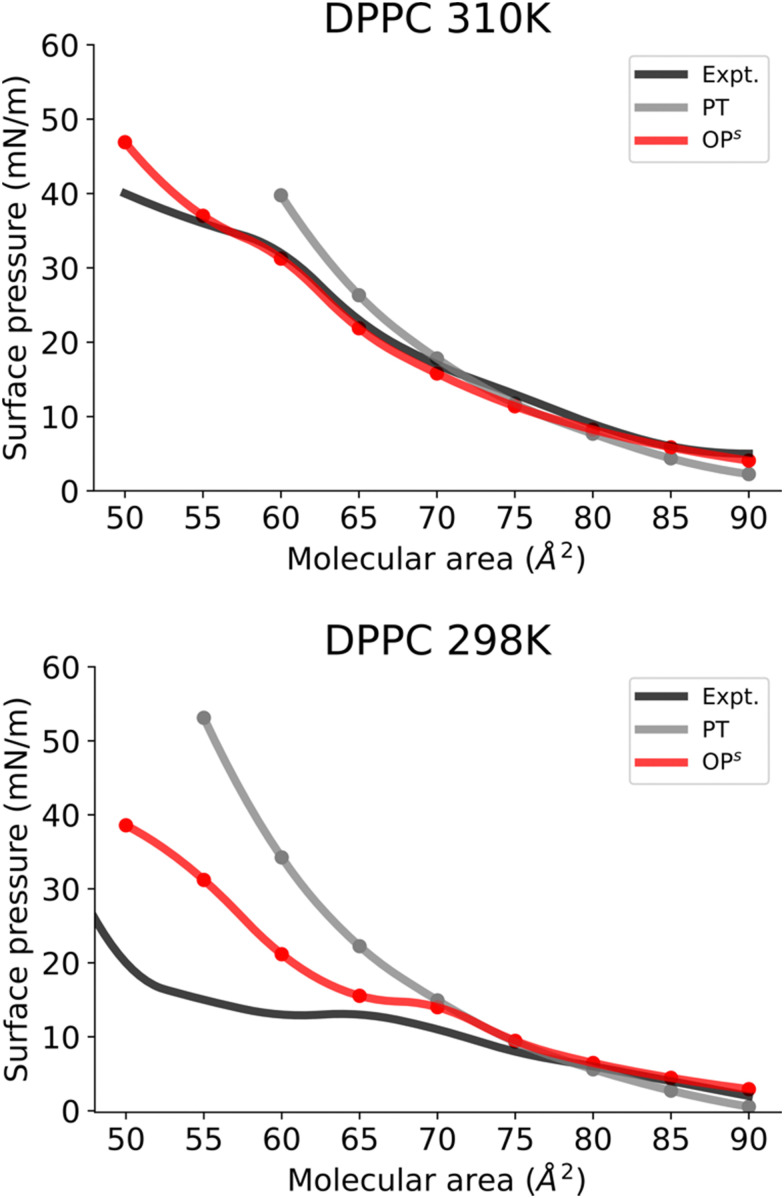
Surface pressure–molecular area isotherms of (A) DPPC and (B) DPPC lipid monolayers at the aqueous sub-phase at 310 and 298 K, respectively. *Label scheme*: Expt. for the experimental *Π*–*A* isotherm, PT for the *Π*–*A* isotherm obtained using the pressure tensor method and OP^s^ for the *Π*–*A* isotherm obtained using the current osmotic pressure method. Error bars are included.

At 310 K, the DPPC monolayer exhibits a transition to the LC phase (Fig. 5A). In accordance with earlier experimental observations,^11^ our MD simulations using the osmotic pressure-based method also reveals a distinct plateau between 55 and 60 Å^2^, with a surface pressure of about 31 mN m^−1^. Furthermore, system snapshots show both ordered and disordered domains in the plateau area as shown in Fig. S5 (ESI†). Fig. 5A clearly shows that the osmotic pressure-based approach yields a qualitatively superior result than the pressure tensor-based approach at low molecular areas. Moreover, the pressure tensor-based simulations proved unstable at a molecular area of 55 Å^2^ and lower.

Next, we investigated the DPPC monolayer at 298 K (Fig. 5B). Here a distinct transition to the LC phase and an area of coexistence between the LC and LE phase at 298 K can be observed (Fig. 5B). The phase coexistence manifests as a plateau in the surface pressure isotherm, with a molecular area value of 65–70 Å^2^ and a surface pressure value of 13 mN/m, consistent with a previous report based on AA simulations.^11^ Fig. S6 (ESI†) shows simulated snapshots from this plateau region that clearly show ordered and disordered regions. Furthermore, Fig. 5B also demonstrates that the osmotic pressure-based method enables us to reproduce all phases for DPPC monolayers qualitatively and far better than the pressure tensor method. Surface pressures just below the phase coexistence region (areas 65–50 Å^2^) are significantly overestimated in the osmotic pressure-based method. This discrepancy probably arises from limitations of the CG model of DPPC at this temperature as this model was developed for the LE phase at 323 K by Shinoda *et al.*^39^ Consequently, this DPPC model is unable to reproduce surface pressure values of a monolayer in the LC phase (at 298 K) quantitatively. Furthermore, we also simulated the isotherms for DPPC in the LE phase at 323 K (Fig. S7, ESI†). The obtained isotherm shows (Fig. S7, ESI†) very good reproduction of the experimental isotherm.

**Fig. 6:**
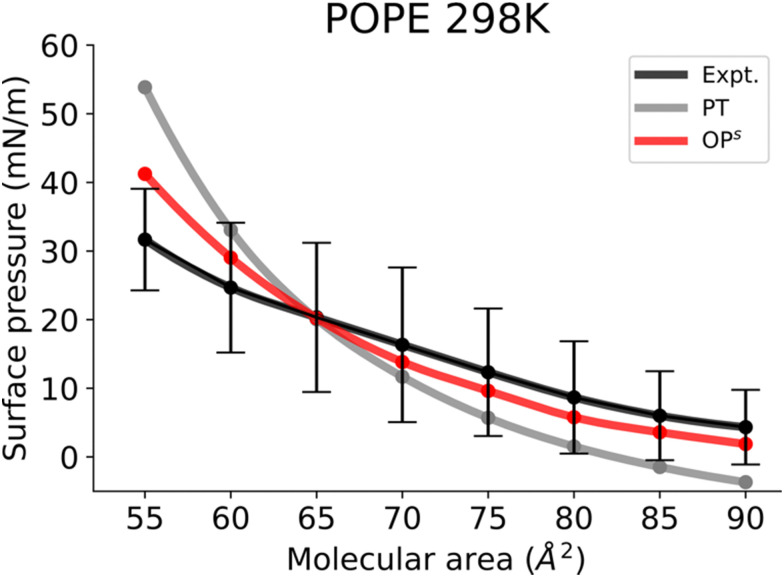
Surface pressure–molecular area isotherms of POPE lipid monolayers at the aqueous sub-phase at 298 K. Label scheme: Expt. for the experimental *Π*–*A* isotherm, PT for the *Π*–*A* isotherm obtained using the pressure tensor method and OP^s^ for the *Π*–*A* isotherm obtained by the current osmotic pressure method. Experimental error bars are included.

Finally, Fig. 6 shows the *Π*–*A* isotherms of POPE monolayers. The simulated isotherms obtained by the osmotic pressure-based method are generally in good agreement with experiments considering the variability of isotherms reported in different experimental reports.^56–58^ The POPE isotherm is characterized by a liquid expanded (LE) phase at a large molecular area. The kink at the onset of the LE−LC phase coexistence is less marked,^56^ and the curve gradually shifts toward higher pressures with compression of the monolayer. Similar to the POPC and DOPC isotherms, the POPE isotherm also exhibits larger surface pressures, and again a marked kink in the surface pressure at the onset of LE−LC phase coexistence. The kink occurs at a much higher surface pressure than in DPPC monolayers. This is because monolayers of unsaturated lipids are less compressible than saturated ones. In the pressure tensor approach, slightly negative surface pressures are seen above the molecular area of 85 Å^2^. The small negative surface pressures imply metastability in the system. However, the *Π*–*A* isotherm obtained from the osmotic pressure method captured the experimental behaviour at high molecular areas too, suggesting that this method is also useful for capturing the gas phase behaviour.

## Conclusions

In this work, we have simulated several phosphatidylcholine (PC) and phosphatidylethanolamine (PE) lipids with classical pressure tensor and osmotic pressure methods to obtain *Π*–*A* isotherms. Our results demonstrate that the *Π*–*A* isotherms calculated using our modified osmotic pressure method are significantly better than the pressure tensor method at lower lipid molecular areas, and they are in excellent agreement with experiments. The osmotic pressure method also proves advantageous compared to the pressure tensor-based methodology by allowing for a single simulation for a given molecular area as compared to two simulations from a pressure tensor-based method. Additionally, the virtual walls allow for easy mobility of the system, allowing for MD simulations and calculation of the surface pressure–area isotherm with a single system. This is advantageous against the set-up of individual systems and MD simulations implementing the pressure tensor-based method for each corresponding molecular area. Thus, the new approach reported in this work paves a new way to extract complete *Π*–*A* isotherms, which can also allow the observation of molecular packing in monolayers at a low lipid molecular area.

Furthermore, we foresee that our methodology will be useful from a computational perspective as this allows appropriate comparison between experiments and simulations, leading to opportunities for parameterization strategies of computational molecular force fields. Subsequently, it may help for physical chemistry researchers to validate their regular solution monolayer model using statistical mechanics, as in ref. 42.

It is worthwhile remarking that eqn (7) is an approximation for a single component monolayer which cannot be regarded as strictly generalized. However, possible extension of this approximation for mixed monolayers where both components form soluble monolayers, or when one component forms an insoluble monolayer while the other is soluble, can be envisaged. This extension does not call for any novel new approach, as it may require only the appropriate definition and calculation of *ξ* for mixed monolayers according to their interactions.

## Author contributions

JP and APS: simulations, analysis, and writing – original draft; APS and SV: conceptualization; SV: supervision and writing – original draft.

## Conflicts of interest

There are no conflicts to declare.

## Supplementary Material

SM-019-D2SM01419J-s001
