# Supplementary material for: An in silico osmotic pressure approach allows characterization of pressure–area isotherms of lipid monolayers at low molecular areas
Source: Soft Matter. 2023 Apr 10;19(18):3377–85. doi: 10.1039/d2sm01419j (PMC10170484; doi:10.1039/d2sm01419j)
Supplement: SM-019-D2SM01419J-s001 [file SM-019-D2SM01419J-s001.pdf]

Electronic Supplementary Material (ESI) for Soft Matter.
This journal is © The Royal Society of Chemistry 2023

Supporting Information for

An in silico osmotic pressure approach allows to characterize pressure-area isotherms of lipid monolayers at low molecular areas

Janak Prabhu#, Akhil Pratap Singh# and Stefano Vanni*

Chemin du Musée 10, Department of Biology, University of Fribourg, Switzerland

Phone: +41-26-300-8896. E-mail: stefano.vanni@unifr.ch

#J.P. and A.P.S. contributed equally to this work and share first authorship.

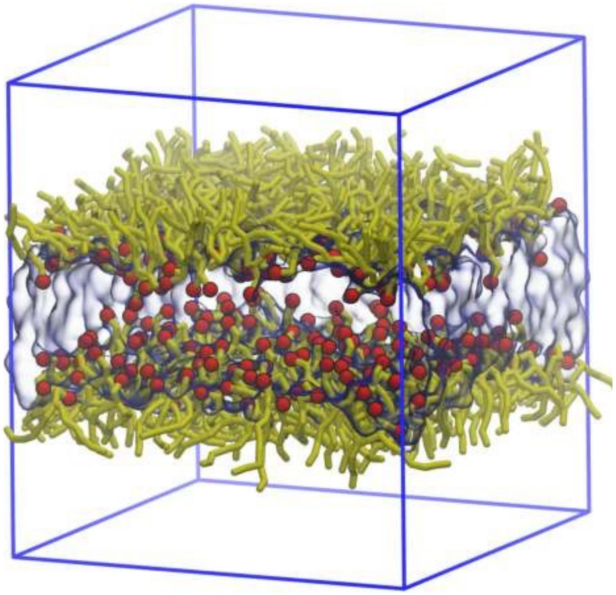

Figure S1. Snapshot of the simulation box for POPC-lipid monolayer. Color scheme: red for lipid head groups, yellow for lipid tails, and ice blue for water

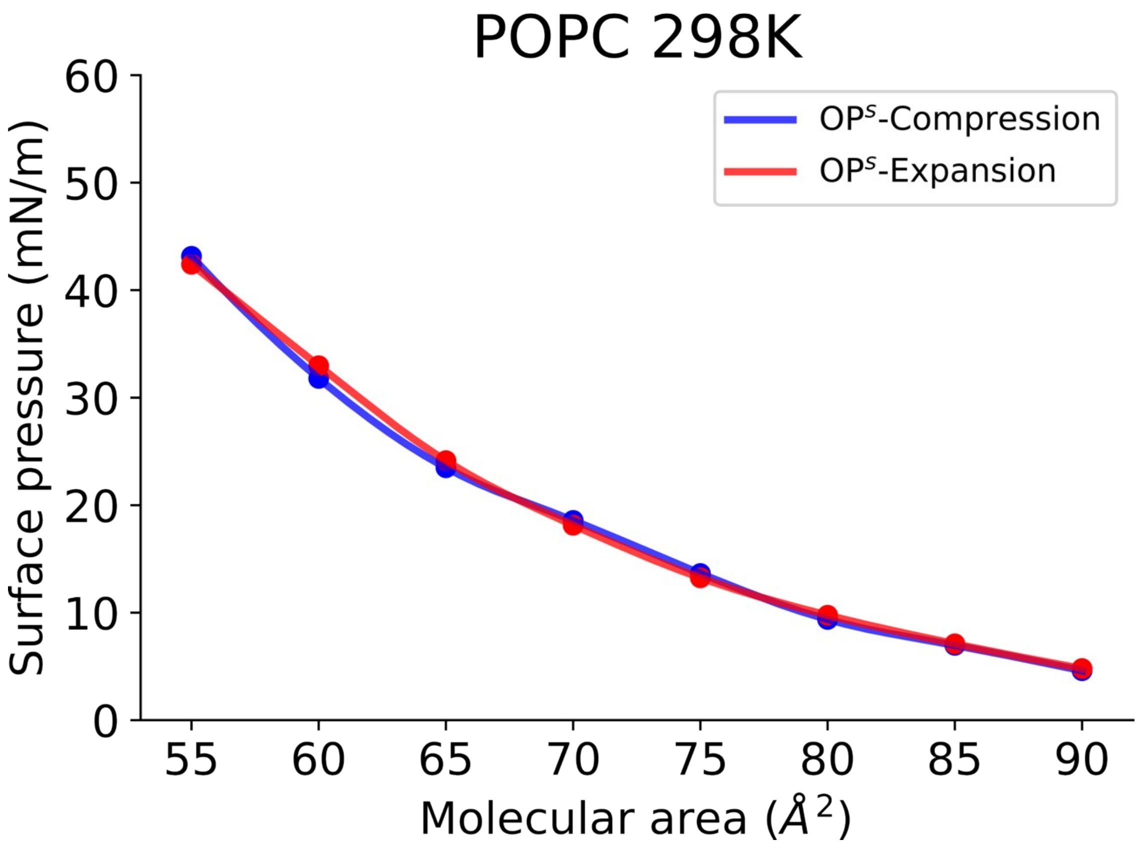

Figure S2. Surface pressure-molecular area isotherms of CG POPC lipids monolayer on aqueous sub-phase at 298 K for different schemes.

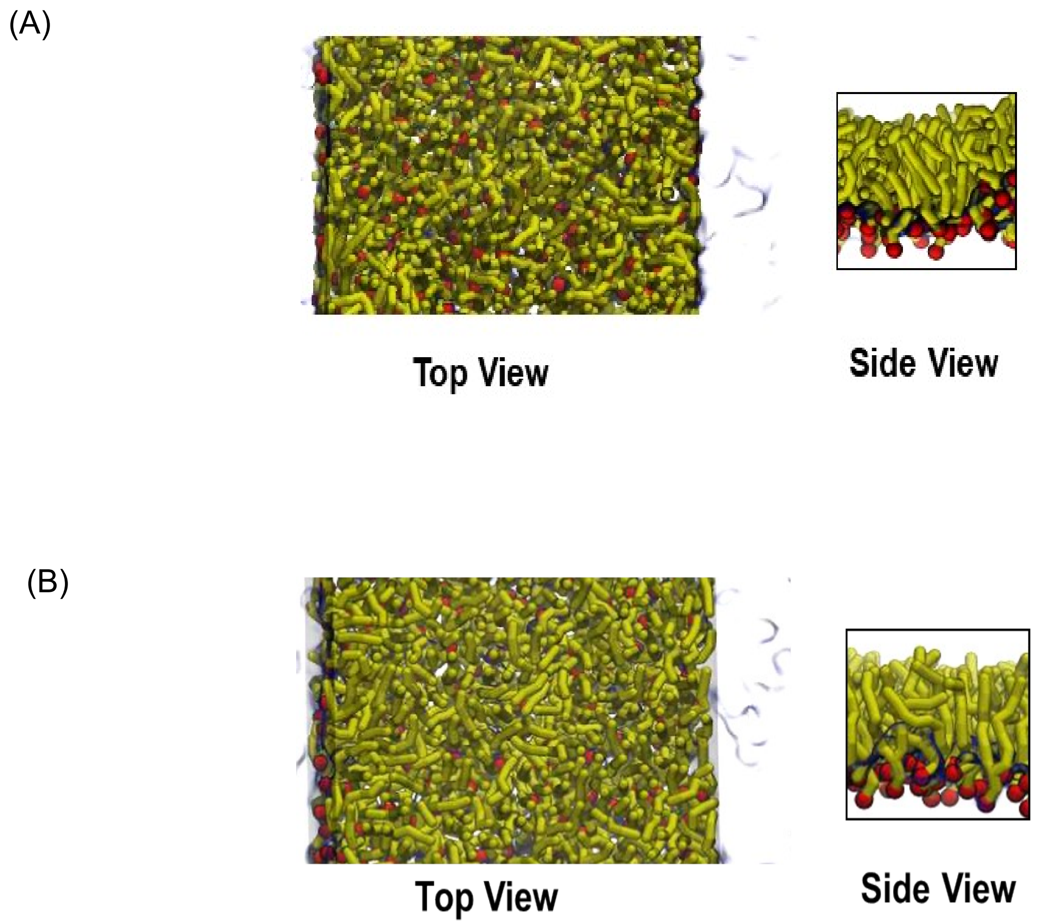

Figure S3. MD Snapshots from simulations using the osmotic pressure method, top and side views, show the liquid-expanded (L_E) phases in (A) POPC and (B) DOPC-lipid monolayers. Color scheme: red for lipid head groups and yellow for lipid's tails.

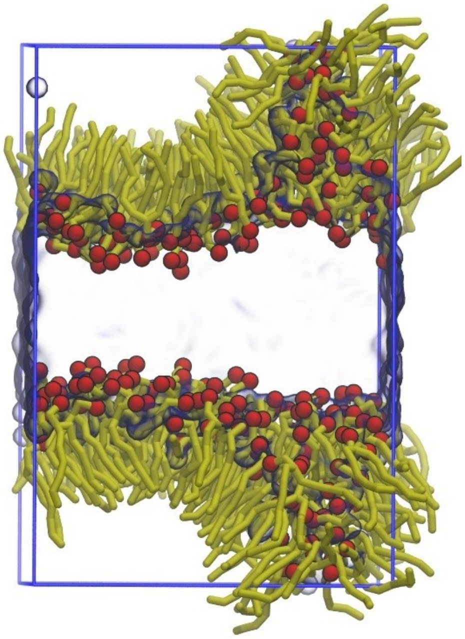

Figure S4. MD Snapshot from simulation using the pressure tensor method shows the unstable DPPC-lipid monolayer at molecular area ~ 50 Å^2. The color scheme is similar to Fig. S1.

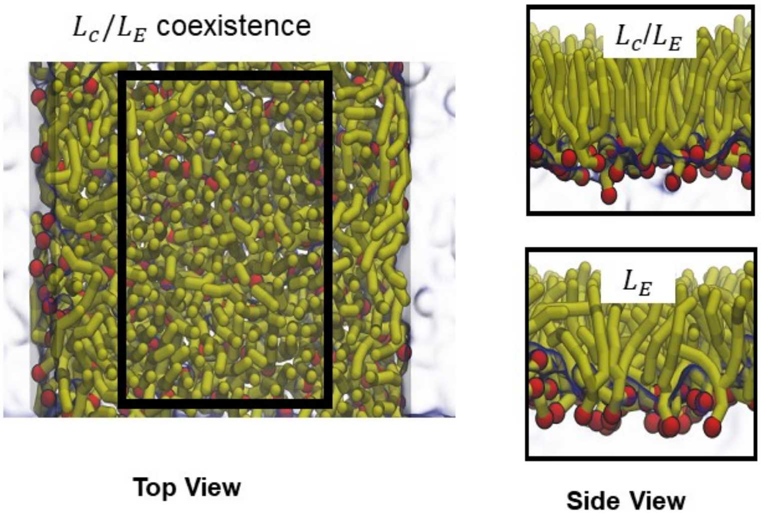

Figure S5. MD Snapshot from simulations using the osmotic pressure method clearly shows the phase coexistence region (L_C/L_E phase) in DPPC monolayers at 310K (molecular area ~ 60 Å^2). Side view snapshots show the difference between a disordered L_E phase and quasi-ordered L_C/L_E phase. The color scheme is similar to Fig. S2.

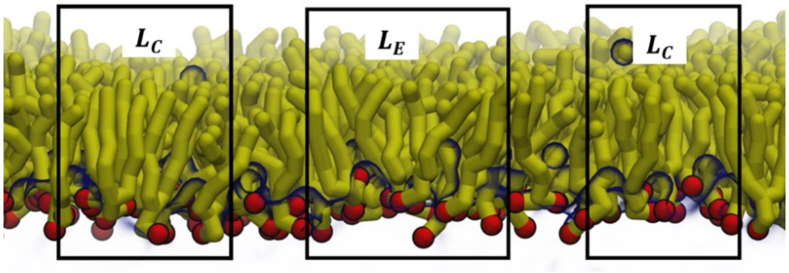

Figure S6. DPPC monolayer side view snapshot at 298K, from simulation using the osmotic pressure method, clearly show the coexistence of disordered (L_E) and ordered (L_C) phase close to phase transition point (molecular area ~ 65 Å^2). The color scheme is similar to Fig. S2.

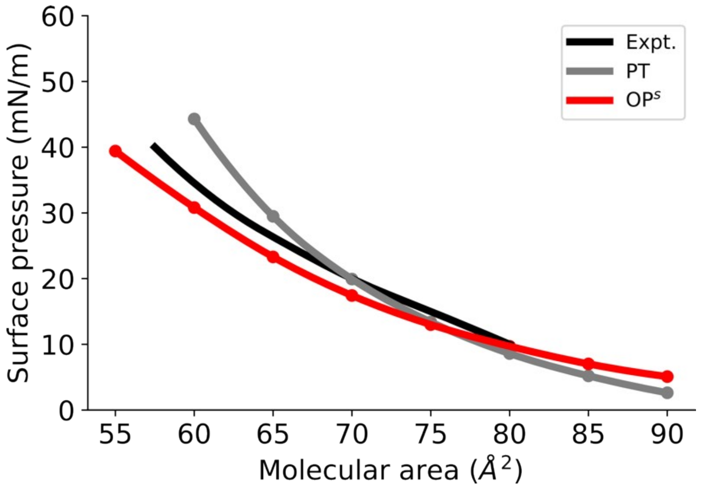

Figure S7. Surface pressure area isotherm of DPPC at 323 K. *Label scheme*: Expt. for experimental Π-A isotherm, PT for Π-A isotherm obtained by pressure tensor method and OP^s for Π-A isotherm obtained by the current osmotic pressure method.

Table S1. Details about simulated lipid monolayer systems to obtain Π-A isotherms by pressure tensor method

**Molecular area ($Å^2$)**	**Lipids per Leaflet/Total**	**Box size in x and y directions (Å)**
50	128/256	80.00
55	128/256	84.00
60	128/256	87.60
65	128/256	91.20
70	128/256	94.60
75	128/256	98.00
80	128/256	101.20
85	128/256	104.30
90	128/256	107.30

Table S2. Details about simulated lipid monolayer systems to obtain Π-A isotherms by Osmotic pressure method

**Molecular area (Å²)**	**Length between virtual walls in x-direction (Å)**	**Length in y-direction (Å)**
50	80	80
55	88	80
60	96	80
65	104	80
70	112	80
75	120	80
80	128	80
85	136	80
90	144	80

Calculation of the semi-empirical coefficient (ξ)

To calculate the semi-empirical coefficient, we first obtained the interfacial depth for lipids at large area per molecule (at infinite dilution). To do so, we implemented the following best exponential curve fitting function to fit the $L_{z;int}$ *vs.* Molecular Area:
The fitting function we implement is given as;

$$L_{z;int} = e^{a/x} + b. \tag{1}$$

Where, x is the molecular area. The fitting parameters are given as a and b. The asymptote of the function is given as,

$$L_{z;int}\,|_{x\to\infty} = b + 1. \tag{2}$$

The factor ξ for a given molecular area (x) is;

$$\xi = \frac{L_{z;int}\,|_{x\to\infty}}{L_{z;int}}. \tag{3}$$

Below Fig. S8 provides an example of for the fitting of POPC at 298 K for the SPICA FF. Similar fits were carried out for rest of the lipids and the coefficients were calculated accordingly.

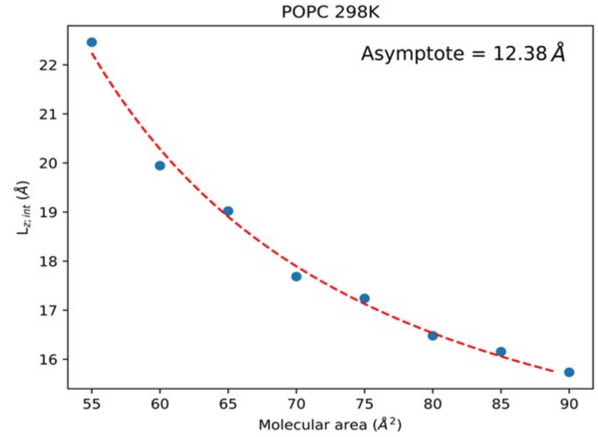

Figure S8. Fitting $L_{Z,int}$ data (blue dots) with the exponential function (eqn. (1); dotted red line) to calculate the asymptote, which is then implemented to obtain the semi-emperical coefficient (ξ) for each molecular area of POPC lipid according to the eq.3.
